# A Review of Parathyroid Surgery for Primary Hyperparathyroidism from the United Kingdom Registry of Endocrine and Thyroid Surgery (UKRETS)

**DOI:** 10.1007/s00268-020-05885-5

**Published:** 2020-12-02

**Authors:** H. Ishii, M. J. Stechman, J. C. Watkinson, S. Aspinall, D. S. Kim

**Affiliations:** 1 Department of ENT, Head & Neck Surgery, St George’s Hospital, Blackshaw Road, Tooting, London, SW17 0QT UK; 2 Department of Endocrine Surgery, University Hospital Wales, Cardiff, UK; 3 Department of Paediatric Surgery, Great Ormond Street Hospital, London, UK; 4 Department of General Surgery, Aberdeen Royal Infirmary, Aberdeen, UK

## Abstract

**Background:**

The United Kingdom Registry of Endocrine and Thyroid Surgeons is a national database holding details on > 28,000 parathyroidectomies.

**Methods:**

An extract (2004–2017) of the database was analysed to investigate the reported efficacy, safety and use of intra-operative surgical adjuncts in targeted parathyroidectomy (tPTx) and bilateral neck exploration (BNE) for adult, first-time primary hyperparathyroidism (PHPT).

**Results:**

50.9% of 21,738 cases underwent tPTx. Excellent short-term (median follow-up 35 days) post-operative normocalcaemia rates were reported overall (tPTx 96.6%, BNE 94.5%, *p* < 0.05) and in image-positive cases (tPTx 96.7%, BNE 96%, *p* < 0.05). Intra-operative PTH improved overall normocalcaemia rates (tPTx 97.8% vs 96.3%, BNE 95% vs 94.4%: both *p* < 0.05). Intra-operative nerve monitoring reduced vocal cord (VC) dysfunction in image-positive tPTx, but not in BNE (97.8% vs 93.2%, *p* < 0.05). Complications were higher following BNE (7.4% vs 3.8%, *p* < 0.05), especially hypocalcaemia (5.3% vs 2%, *p* < 0.05). There was no difference in rates of subjective dysphonia following tPTx or BNE (2.4% vs 2.3%, *p* > 0.05), nor any difference in VC dysfunction when formally examined (4.9% vs 4.1%, *p* > 0.05).

**Conclusions:**

In image-positive, first time, adult PHPT cases, tPTx is as safe and effective as BNE, with both achieving excellent short-term results with minimal complications.

## Introduction

The United Kingdom Registry of Endocrine and Thyroid Surgery (UKRETS) is a multi-centre, multi-disciplinary (General, Otolaryngology, Oral and Maxillofacial, Transplant and Vascular surgeons) database. It is the world’s largest endocrine surgery database and is maintained by the British Association of Endocrine and Thyroid Surgeons (BAETS) members.

UKRETS has recognised potential deficiencies in care, including under-diagnosis and heterogeneous practise across the United Kingdom (UK). Therefore, the National Institute for Health and Care Excellence (NICE) have published guidelines on the management of primary hyperparathyroidism (PHPT) in the UK [1].

Targeted parathyroidectomy (tPTx) in selected patients, with pre-operative localisation and intra-operative parathyroid hormone (ioPTH), have led to well-documented reductions in operating time and post-operative complications (especially hypocalcaemia) [2–5]. However, there is the potential to miss multiglandular disease (MGD) [6, 7], leading to recurrences. ioPTH during tPTx may mitigate this risk, but its benefit in first-time parathyroidectomy remains debatable due to its cost, only moderately high specificity in identifying those cases with remaining abnormal tissue following adenoma excision [8], and marginal improvement in cure [5]. A literature review by Morris et al. [9] demonstrated that in a case of localised PHPT, ioPTH increased the success rate of tPTx from 96.3 to 98.8%, whilst incurring an approximate additional cost of 4% to the procedure. However, the authors did also conclude that there were institution specific factors that influenced the value of ioPTH.

With this in mind, a review of parathyroid surgery recorded in the UKRETS was undertaken.

## Aims

This study investigated the recorded efficacy and safety of tPTx and bilateral neck exploration (BNE) with and without surgical adjuncts in first-time PHPT cases from the UKRETS.

## Material and methods

Access to UKRETS was granted through the BAETS executive committee in February 2018, for data from 2004 to 2017. Patient consent is obtained by surgeons to collect and anonymously analyse data in UKRETS along General Data Protection Regulation requirements. Clinical data are uploaded by surgeons, but the dataset has not been externally validated. The data fields for parathyroid surgery are available in the appendix of the Fifth National Report [9]. The lead author (HI) analysed the database.

Adults (≥ 18 years old) undergoing first-time parathyroidectomy for sporadic PHPT between 2004 and 2017 were included. Cases with illogical/inaccurate data (e.g. age > 100 years old) and cases not specified as first-time surgeries were excluded. Cases with incomplete outcome data were excluded from specific sub-analyses to prevent skewing of interpretation. Missing data rates varied across outcomes, so the denominator (total number of cases analysed) varied depending on the outcome in question.

The recorded surgical technique (tPTx or BNE), adjuncts and number of glands removed were analysed. tPTx cases converted to BNE were analysed as BNE. Complication rates and their nature were analysed. The method (US, indirect or direct laryngoscopy) by which formal vocal cord (VC) function was examined is not specified in UKRETS.

UKRETS does not record operating centre or operative findings, therefore, it was not possible to explain why (failure to identify adenoma, identifying two ipsilateral adenomas, or failure of ioPTH to fall below 50% of baseline) some tPTx cases were converted to BNE. Neither does it record decision-making on ioPTH or ioNM use, availability of ioPTH/ioNM or operative approach (tPTx or upfront BNE). Therefore, no comments or inferences on these topics were made.

## Definitions

BAETS [8] defines tPTx as “*surgery that may include mini-incision open techniques, endoscopic methods or even unilateral neck exploration (UNE)*”, but UKRETS does not distinguish between these techniques.

“*Image-positive*”cases where pre-operative localisation modalities (ultrasonography (US) and/or nuclear medicine (NM) and/or computed tomography (CT)/magnetic resonance imaging (MRI)) identified target gland(s). UKRETS does not record of the number or location(s) of the target gland(s).

“*Hypocalcaemia*” low serum corrected calcium < 2.1 mmol/L or ionized serum calcium < 1.2 mmol/L at first post-operative day.

“*Normocalcaemia*” was defined as the absence of hypocalcaemia and persistent hypercalcaemia at the first post-operative visit.

“*Subjective dysphonia*” the subjective (assessed by the surgeon) presence of voice change at the first post-operative visit.

## Statistical analysis

Descriptive statistics are reported as medians with interquartile ranges (IQR; 25–75%), unless stated otherwise. Medians were used to avoid central tendencies being affected by outlying values.

Differences between groups and categorical variables were calculated with the Mann–Whitney U and Fisher’s exact test, respectively. *P*-values of < 0.05 were considered statistically significant.

GraphPad Prism, version 8.0.1 (GraphPad Software, 7825 Fay Avenue, Suite 230, La Jolia, CA 92,037, USA) was utilised for statistical analyses and Microsoft Excel, version 16.12 was used for data handling.

## Results

**Fig. 1:**
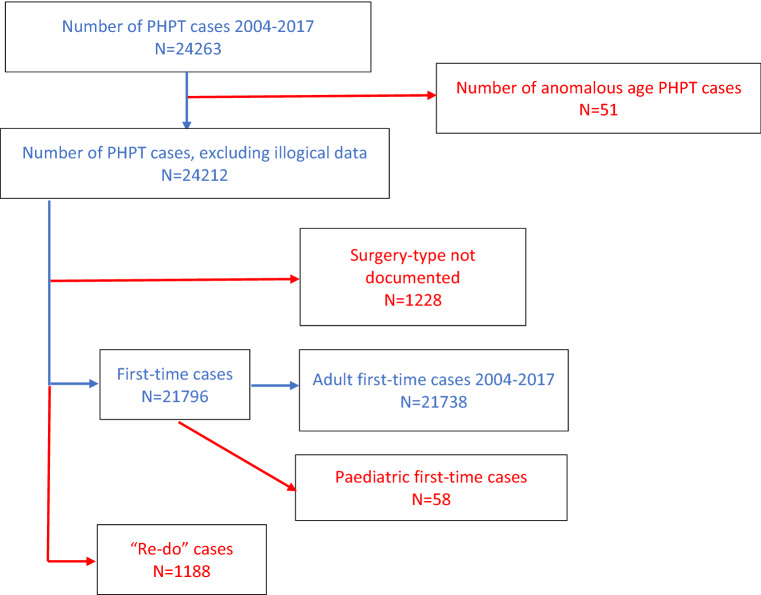
Flow diagram of the United Kingdom Registry and Endocrine and Thyroid Surgery database

**Fig. 2:**
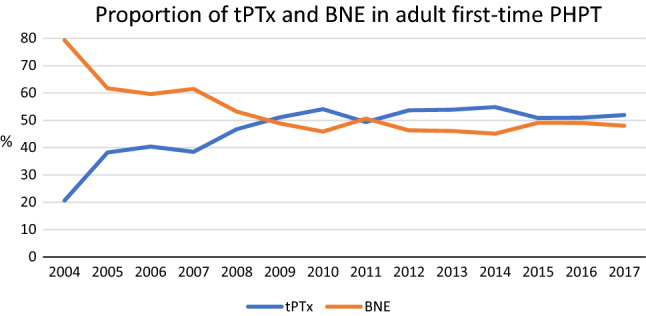
Proportion of tPTx and BNE in UKRETS between 2004 and 2017 for PHPT

After applying exclusions, there were 21,738 first-time PHPT cases between 2004 and 2017 (Fig. 1); 50.9% (*n* = 11,062) underwent tPTx and 49.1% (*n* = 10,676) BNE. 8.1% (867/10,676) were converted from tPTx to BNE. Between 2004–07, BNE was most frequently utilised, but since 2008, both techniques have been employed equally (Fig. 2).

BNE and tPTx were performed in 52.2% (5570/10,676) and 98.1% of image-positive cases (10,850/11,062), respectively. It was not possible to analyse how many of the converted cases were due to a failure of ioPTH levels to drop, as UKRETS did record this. Overall, ioPTH was used more frequently during tPTx (25%; 63/11,602) than BNE (19.1%, 2044/10,676) (*p* < 0.05). ioPTH use has risen from 0% (0/20) of tPTx and 1.3% (1/77) of BNE in 2004 to 29.8% (354/1187) and 28.6% (317/1098) in 2017, respectively.

On average, 1.25 glands were removed in image-positive BNEs, compared to 1.07 in image-positive tPTx and 1.34 glands in all (image-positive and -negative) BNEs (Table 1).

**Table 1:** Number of parathyroid glands removed per parathyroidectomy

No. of glands removed	Image-positive BNE (total *n* = 5570)	Image negative BNE (total *n* = 4029)	Image-positive tPTx (total *n* = 10,850)
0	166 (3.0)	107 (2.7)	58 (0.5)
1	4043 (72.6)	2484 (61.7)	9916 (91.4)
2	1063 (19.1)	871 (21.6)	667 (6.1)
3	181 (3.2)	388 (9.6)	70 (0.6)
3.5	51 (0.9)	93 (2.3)	33 (0.3)
4	20 (0.4)	39 (1.0)	11 (0.1)
Total number of glands removed	6971	5872	11,620

Values in parentheses are percentages

ioNM use was documented in 3151 cases: 17.5% of tPTx (1732/9851) versus 15.4% of BNE cases (1419/9198) (*p* < 0.05). By 2017, ioNM was used in 34.1% of tPTx (405/1187) and 29.8% of BNE cases (327/1098). There were 1296 parathyroidectomies (6%; 1296/21,738) where ioNM use was documented with a formal post-operative VC functional outcome. ioNM was NOT used, but with a formal post-operative VC functional outcome in 2209 parathyroidectomies (10.2%; 2209/21,738). More cases had normal post-operative VC function following tPTx with ioNM (97.8%; 657/672), than tPTx without ioNM (93.2%; 1112/1193) (*p* < 0.05). There was no difference in VC function with or without ioNM in BNE cases (Table 2). This group represents only 6% of the dataset due to the infrequent use of ioNM and incomplete datasets.

**Table 2:** Rates of normal vocal cord movement following tPTx and BNE in UKRETS with and without ioNM

	With ioNM	Without ioNM	*P* value
	*N* (%)		
Image-positive tPTx	657/672 (97.8)	1112/1193 (93.2)	< 0.05
Image-positive BNE	386/398 (97)	576/604 (95.4)	NS
Image negative BNE	219/226 (97)	398/412 (96.7)	NS
Total no. of cases	1296/21,738 (6)	2209/21,738 (10.2)	< 0.05

There was no difference in rates of subjective dysphonia following tPTx or BNE (2.4%; 219/9003 vs 2.3%; 212/9093, *p* > 0.05). There was no difference between tPTx and BNE in VC dysfunction when formally examined (4.9%, 93/1890 vs 4.1%, 70/1716, *p* > 0.05) (Table 3). Formal VC examination was either not performed or not recorded in most cases (83.4%; 18,132/21,738).

**Table 3:** Incidence and missing data for subjective dysphonia and abnormal VC assessment following parathyroidectomy, comparing outcomes from tPTx and BNE

	tPTx (%)	BNE (%)	*P* value
Subjective dysphonia	219/9003 (2.4)	212/9093 (2.3)	NS
Post-op VC check done	1890/11,062 (17.1)	1716/10,676 (16.1)	NS
Post-op abnormal VC check	93/1890 (4.9)	70/1716 (4.1)	NS

The median time to first post-operative visit was 35 days (IQR 19–51). Normocalcaemia rate was higher following tPTx (96.6%; 8375/8664) than BNE (94.5%; 8254/8735) (*p* < 0.05). Normocalcaemia following tPTx was higher with ioPTH (97.8%; 2291/2343) versus without ioPTH (96.3%; 6084/6321) (*p* < 0.05). There was no difference in normocalcaemia rates following BNE with or without ioPTH (95.0%; 1603/1688 vs 94.4%; 6651/7047, *p* > 0.05) (Table 4).

**Table 4:** Rate of normocalcaemia following parathyroidectomy comparing tPTx and BNE with and without ioPTH in overall and image-positive cases

	tPTx (%)	BNE (%)	*P* value
Overall	8375/8664 (96.6)	8254/8735 (94.5)	< 0.05
With ioPTH	2291/2343 (97.8)	1603/1688 (95.0)	< 0.05
Without ioPTH	6084/6321 (96.3)	6651/7047 (94.4)	< 0.05

	Image + ve tPTx (%)	Image + ve BNE (%)	
Overall	8126/8397 (96.7)	4131/4305 (96.0)	< 0.05
With ioPTH	2200/2249 (97.8)	730/760 (96.1)	< 0.05
Without ioPTH	5926/6148 (96.4)	3401/3545 (96.0)	NS

Image-positive tPTx had higher normocalcaemia rates than BNE (96.7%; 8126/8397 vs 96.0%; 4131/4305) (*p* < 0.05) and especially when ioPTH was used (97.8%; 2200/2249 vs 96.1%; 730/760) (*p* < 0.05). There was no difference in normocalcaemia rates between image-positive tPTx (96.4%; 5924/6148) and BNE (96.0%; 3401/3545) without ioPTH. Normocalcaemia rates following BNE overall were lower than those in image-positive BNE (94.5%; 6651/7047 vs 96.0%; 3401/3545) (*p* < 0.05) (Table 4). Missing data represented 17.4% (3430/21,738) of cases.

Overall, BNE had a higher complication rate than tPTx (7.4%; 786/10,676 vs 3.8%; 417/11,062) (p < 0.05), mostly due to hypocalcaemia (5.3%; 533/10,131 vs 2.0%; 211/10,360) (Table 5).

**Table 5:** Overall complications following tPTx and BNE

Complications	tPTx (%)	BNE (%)	P value
Overall complication rate	417/11,062 (3.8)	786/10,676 (7.4)	< 0.05
Wound infection	28/11,062 (0.3)	47/10,676 (0.4)	< 0.05
Haematoma requiring RTT	36/10,505 (0.3)	44/10,195 (0.4)	NS
Hypocalcaemia (NOS)	211/10,360 (2.0)	533/10,131 (5.3)	< 0.05
Other complications	142/11,062 (1.3)	162/10,676 (1.5)	NS

*NS* not significant, *NOS* not otherwise specified, *RTT* return to theatre

Image-positive BNE had higher complication rates than image-positive tPTx (4.9%; 274/5570 vs 3.5%; 376/10,850) (*p* < 0.05). Wound infection and hypocalcaemia rates were lower following image-positive tPTx compared to image-positive BNE (Table 6). Analysis of image-positive cases where only one gland was removed that resulted in hypocalcaemia demonstrated tPTx had a lower rate that BNE (2.6%; 159/6062 vs 3.5%; 141/4043, *p* < 0.05).

**Table 6:** Complications following parathyroidectomy in image positive cases

Complications	Image + ve tPTx (%)	Image + ve BNE (%)	*P* value
Overall complication rate	376/10,850 (3.5)	274/5570 (4.9)	< 0.05
Wound infection	23/10,850 (0.2)	26/5570 (0.5)	< 0.05
Haematoma requiring RTT	36/10,310 (0.3)	14/3357 (0.4)	NS
Hypocalcaemia (NOS)	203/10,188 (2.0)	165/3335 (4.9)	< 0.05
Other complications	114/10,850 (1.1)	69/5570 (1.2)	NS

Of 744 hypocalcaemic cases, 434 (58.3%) had records of whether or not vitamin D/calcium supplements were still being taken at the first post-operative visit. Fewer tPTx cases were taking supplements compared to BNE cases (13.1%; 18/137 vs 24.3%; 72/296, *p* < 0.05). Unfortunately, there is no long-term hypocalcaemia data.

21.2% of tPTxs (2068/9838) were discharged on the same day, versus 11.2% of BNEs (1089/9787) (*p* < 0.05). There was no difference in discharge frequency on the first post-operative day (tPTx 66.2%; 6514/9838 vs BNE 66.4%; 6500/9787, *p* > 0.05). Length of stay greater than one day was more common in BNE than tPTx (22.5%; 2198/9787 vs 12.8%; 1256/9838) (*p* < 0.05).

## Discussion

This analysis of 21,738 adult first-time PHPT cases between 2004 and 2017 is the largest reported multi-centre, multidisciplinary review of parathyroid surgery in the world. 11,602 (50.9%) underwent tPTx, comparable to other registry studies; Kazaure et al. [10] (55.9%, *n* = 3592) and Bergenfelz et al. [11] (39%, *n* = 1050).

Despite positive pre-operative localisation, 52% of patients underwent BNE. 8% of these (867/10,676) were initially treated as tPTx then converted to BNE, inferring that 44% of image-positive cases underwent upfront BNE. It was not possible to report if cases underwent BNE due to multiple glands being identified on pre-operative localisation, which may explain why a high proportion of cases underwent BNE with positive localisation. Other potential hypotheses include surgeon preference on operative technique, variable imaging quality and patient factors (high body mass index) and shared decision-making.

Normocalcaemia rates following both techniques were excellent and are comparative to reports by Jinih et al. [12] (tPTx 96.4%, BNE 96.7%; no clear definition of “cure”) and Ospina et al. [13] (tPTx 98%, BNE 97%; variable definition of “cure”). The authors acknowledge that “*normocalcaemia*” in this study does not equate to the American Association of Endocrine Surgeons (AAES) definition of “cure” [2]. The UKRETS does not currently collect data on long-term (more than six months) follow-up data. Normocalcaemia rates for image-positive cases suggestive of single gland disease managed by tPTx and BNE (96.7% vs 96.0%), although statistically significant, were marginal and so of questionable clinical importance. This supports the recommendation of discussing both operative approaches in image-positive patients [1].

Normocalcaemia rates following tPTx were excellent with (97.8%) or without (96.4%) ioPTH. These figures underestimate the benefit of ioPTH, as cases converted from tPTx to BNE, possibly due to failure of ioPTH to fall below 50% of baseline, were analysed as BNE, and so not included in the analysis of tPTx. Ishii et al. [14] reported overall cure at six months was higher (99.3% vs 98.1%, *p* < 0.05) and recurrence lower (0.2% vs 1.5%, *p* < 0.05) when ioPTH was utilised with minimally invasive parathyroidectomy. They found ioPTH was less likely to be utilised in image concordant (versus image discordant) cases. The low record of ioPTH in UKRETS suggests it is still not being routinely used. However, the database demonstrated that the rate of ioPTH was at its highest in 2017. NICE [1] does not recommend routine use of ioPTH in first-time surgery, which seems justified, given the extra cost, for only marginal improvements in normocalcaemia rates. Neither NICE or AAES currently recommend routine use of ioNM [1, 2]. UKRETS demonstrated, in a small subset of cases (6%) which reported ioNM use with a documented post-operative VC assessment outcome, there were fewer abnormal VC function following tPTx, but not BNE. It is unclear why ioNM should be beneficial in tPTx but not BNE, though limited dissection during tPTx may compromise a satisfactory view of the recurrent laryngeal nerve. ioNM was used more frequently in tPTx than BNE, probably due to increasing popularity of tPTx coinciding with increasing availability of ioNM. Given the small proportion of cases with reported ioNM use, these results may not be representative of or applicable to overall care. However, UKRETS demonstrated an association between ioNM in tPTx and improved rates of post-operative normal VC function.

Overall and image-positive complication rates were significantly lower following tPTx versus BNE. These figures are similar to Jinih et al. [12] (tPTx 3.7%, BNE 17.1%). Bergenfelz et al.’s [11] review of the SQRTPA database reported overall complication rates of 3.4% (*n* = 92).

It was not possible to ascertain if hypocalcaemia was temporary or permanent or if there was a cause such as hungry bone syndrome. Hypocalcaemia rates were significantly higher following BNE than tPTx, but this study found that more glands were excised during BNE, so this higher rate is expected.

Abnormal VC function following tPTx (4.9%) was higher than Jinih et al.’s [12] and Ishii et al.’s [14] numbers. The high rate of missing data for VC function meant it was difficult to draw any definitive conclusions. Subjective dysphonia rate was 2.4% (no difference between techniques). However, subjective assessment is inaccurate, variable and may potentially underestimate actual VC dysfunction [15, 16]. Rates of formally assessed post-operative abnormal VC function were 4.9% following tPTx and 4.1% following BNE (*p* > 0.05), but it is not fair to compare these figures against subjective dysphonia rates as there may be other reasons for dysphonia (injury to external branch of superior laryngeal nerve, vocal cord oedema, surgical scar tissue, etc.).

BAETS do not endorse same-day discharge following thyroidectomy due to potential airway obstruction secondary to post-operative haematoma [17]. This has influenced parathyroidectomy practise and may explain the similar proportion of overnight stays following tPTx and BNE. BNEs had longer post-operative stays which may be partially explained by the higher hypocalcaemia rate in this group, where normalising calcium may have required intravenous replacements, delaying discharge.

## Strengths and limitations

Registries are useful to answer certain questions that are not suitable for randomized clinical trials. This registry study provides the best insight into parathyroidectomy outcomes across the UK. Multi-disciplinary surgeons have contributed from both small and large volume centres, reducing the risk of skewed data, and allowing comparison with recognised guidelines [1, 2]. The large number of cases reduces the risk of bias/errors that are inherent from small sample sizes, and highlights statistically significant differences between groups with similar clinical outcomes. It is important to consider these results carefully, as the number of patients needed to treat to achieve comparable results may not justify expensive interventions for only marginal benefits.

Limitations include the significant amount of missing data, design flaws of data collection, potential self-reporting bias, coding errors, voluntary nature of uploading cases and lack of external validation (though this is currently being addressed). The results only allowed a median follow-up of 35 days, meaning long-term outcomes (cure, persistent disease and recurrence rates) were not recorded, limiting its use when comparing techniques. “*Targeted parathyroidectomy*” as per UKRETS, encompasses various surgical techniques, which may affect outcomes. Selection criteria for surgery, symptoms, complications or severity of PHPT are not recorded and therefore not assessed.

An example of potential coding errors is highlighted in the number of glands removed by operative technique. An average of 1.07 glands was excised during tPTx, most likely because UNEs are coded as tPTx in UKRETS. It is not possible to explain why 3, 3.5 or 4 glands were excised during tPTx, as UKRETS does not record pre-operative findings or histology of excised glands, though presumably these may be coding errors.

Factors confounding the interpretation of results include the fact that only selected patients underwent tPTx, therefore the incidence of MGD is likely to be higher in cases undergoing BNE, where more glands were excised. This affects comparisons of normocalcaemia and hypocalcaemia rates between tPTx and BNE. However, this study did demonstrate that in image-positive cases where only one gland was removed, BNE had a higher rate of post-operative hypocalcaemia than tPTx.

Interpretation of localisation results are limited. The number anatomical location of pathological glands or whether the results corresponded to operative findings are not recorded. Hence any inferences about the concordance or discordance of localisation modalities cannot be made.

This study has highlighted areas of the registry that can be updated to improve data collection and analysis.

## Conclusions

This study has reported on the largest parathyroid registry in the world; 21,738 cases of first-time primary hyperparathyroidism treated between 2004 and 2017. This study has demonstrated that in image-positive cases, tPTx is as safe and effective as BNE, with both achieving excellent results with minimal complications.
